# Association between maximal lower leg strength and static and dynamic balance as well as gait velocity in older adults

**DOI:** 10.3389/fragi.2026.1736517

**Published:** 2026-03-30

**Authors:** Konstantin Warneke, Andreas Stotz, Andreas Konrad, Astrid Zech

**Affiliations:** 1 Institute of Sport Science, Department for Human Movement Science and Exercise Physiology, Friedrich Schiller University Jena, Jena, Germany; 2 Institute of Human Movement Science, Sport and Health, Universitat Graz, Graz, Austria; 3 Department for Human Movement Science and Exercise Physiology, Universitat Hamburg, Hamburg, Germany

**Keywords:** dynamic balance, elderly, lower-leg strength, static balance, walking speed

## Abstract

**Purpose:**

Falls cause serious injuries with partially long immobilization time and decreased quality of life in older adults. The risk factors comprise instability in gait and balance, which were moderately correlated with the strength capacity. While the literature focused on upper-limb strength, in this work, we sought to evaluate the relationship between maximal plantar flexion and dorsiflexion strength and walking and balance parameters in older adults.

**Methods:**

A total of 51 healthy and active participants (age: 78.5 ± 5.8 years) participated in the study. Lower-leg maximal strength was determined isometrically. Selected gait parameters (normal and maximal walking velocity), static balance [center of pressure (CoP) sway in different standing conditions], and the timed up and go (TUG) and sit-to-stand (STS) were determined. Correlations [Pearson (r_p_)/Spearman (r_s_)] were calculated in general and stratified by sex.

**Results:**

Walking velocity, STS, and TUG were significantly influenced by lower-leg strength (r_s_ up to 0.79 in males). Static balance showed no meaningful relationships. In general, fewer correlations in female participants reached the level of significance and showed smaller effect sizes.

**Discussion:**

Although smaller sex-subgroup sample sizes might limit confidence in the results, male participants showed higher correlations between strength and walking velocity (up to r_s_ = 0.79) and individual balance parameters (r_s_ = 0.77) than female participants (r_s_ = 0.56 for gait, r_s_ = 0.72 for TUG). The results align with previous studies showing a potential influence of strength on gait parameters; however, a causal relationship must be confirmed by longitudinal study designs. Nevertheless, based on the results, there is a need for future sex-specific studies on the necessity of sex-specific balance and fall-prevention routines.

## 1 Introduction

The demographic change is, among others, one of the most challenging socioeconomic burdens societies need to manage today (Cruz-Jentoft et al., 2019). While people reach higher ages due to various factors, unhealthy aging is accompanied by multiple morbidities, which can significantly hamper quality of life (Cruz-Jentoft et al., 2019; Davis et al., 2010). “Intrinsic capacity” is described as a crucial concept of health aging and was described by the World Health Organization (WHO) as *“the composite of all the physical and mental capacities that an individual can draw on at any point in time”* (Bautmans et al., 2022). To counteract age-related intrinsic capacity loss, physical activity is a recommended cornerstone (Distefano and Goodpaster, 2018; Nelson et al., 2007) and has several health benefits, e.g., as a prevention strategy for sarcopenia (Cruz-Jentoft et al., 2019). Both function and structural loss are accompanied by an increased risk of falls—one main cause of femoral bone fractures (Schick et al., 2018) or hip fractures (Florschutz et al., 2015), leading to hospitalization, immobilization, and reduced quality of life (Pernambuco et al., 2012; Sherrington et al., 2011). In the U.S., fatal and non-fatal falls costs in 2015 were approximated at 50.0 billion$ (Florence et al., 2018).

In 2008, the WHO reported that 28%–35% of people over 65 years of age and up to 42% of people over 70 years of age fall once per year (World Health Organization, 2007). To counteract the increasing incidence observed over the years, the WHO called for intensified research efforts to identify predictors to optimize the mobilization of resources for fall prevention strategies in vulnerable populations.

As a multifactorial construct, falls are, however, influenced by a variety of variables, such as medication, osteoarthritis, depression, dizziness, and sarcopenia. The important role of muscle function and structure, however, is expressed in the American Geriatrics Society and American Academy of Orthopedic Surgeons panel guidelines on fall prevention, which list muscular weakness as one of the most important risk factors for falls in older participants (Author Anonymous, 2001). Sarcopenia is described as the age-related function loss causing muscle atrophy and a decrease in strength. This impairment in muscle function and structure can have negative consequences on managing daily tasks. In walking, these factors were significantly related to impaired walking speed, shortened stride length, and increased double support time (Verghese et al., 2009). In addition, the capability to maintain balance during different standing tasks plays an important role in fall prevention (Melzer, 2004), but it declines with aging (Konrad et al., 1999).

In the literature, the participants’ capability to produce higher lower-limb force output was significantly associated with increased balance and gait performance, indicating that both parameters could be important in fall prevention (Cho et al., 2012). In a systematic review with meta-analysis, Muehlbauer et al. (2015) pooled effects (ES) from eight studies and found a small but significant influence of muscle strength on static balance (r = 0.27) and gait speed (r = 0.35), which was considered dynamic steady-state balance. Methodological heterogeneity limits the validity of the generalized conclusions as the authors mixed the effects from different muscle groups, including leg extensor muscle strength (Muehlbauer et al., 2012; Izquierdo et al., 1999) along with foot and ankle strength (Spink et al., 2011) in older adults. This is relevant as the correlations were moderated by muscle location (Shin et al., 2012) and sex (Stotz et al., 2023a).

While most studies focused on hip and thigh muscle strength, showing positive correlations, evidence regarding the effect of ankle strength on postural sway and gait relationships is inconclusive. In 164 older participants, Song et al. (2021) reported trivial relationships between plantar flexor and dorsiflexor strength and the center of pressure (CoP) sway with r = 0.13–0.15. In contrast, Maktouf et al. (2018) found mixed results for the relationship between lower-leg strength and different balance outcomes within the older population, with correlations of up to r = 0.87. An important test to monitor the ability of linear walking, balance, and functional strength is the timed up and go (TUG), which is commonly performed and is not only predictive of sarcopenia (Martinez et al., 2015) [as associated with strength capacity (Benavent-Caballer et al., 2016)] but also of fall recurrence (Kang et al., 2017). In accordance with Jalali et al. (2021) (r = −0.42 to −0.45, *p* < 0.001), Wang et al. (2022) reported plantar flexor strength to be related to TUG performance (r = −0.47). Stotz et al. (2023a) found only gait speed and stance time to be slightly correlated with plantar flexor strength (r = 0.37–0.39).

Since only few studies were performed to examine the relevance of lower-leg muscle strength on static and dynamic balance performance as well as gait abilities, this study was designed to contribute to our understanding of specific muscle strength capacities as a potential contributer. Due to previous review articles, a positive influence of maximal strength on the listed outcomes is hypothesized.

## 2 Methods

The study was designed as exploratory research aimed at identifying future potential for longitudinal designs and generating hypotheses for interventional studies. In accordance with previous literature and its importance as functional tests in older adults, the primary outcomes for this study were the correlations between walking velocity in the 6-m walking test, the TUG, and plantar flexor strength. Static balance in different stance conditions and the sit-to-stand (STS) test stratified by sex were considered secondary outcomes.

### 2.1 Participants

G-Power sample-size estimation was performed for t-tests with a point-biserial correlation model for two-tailed correlations, with α error probability set to 0.05 and 1–β error probability set to 0.8, with gait speed correlation to plantar flexion strength as the primary parameter (r = 0.40) (Stotz et al., 2023a). The smallest sample size was estimated as n = 44. To account for potential drop-outs and unsuccessful trials, 51 healthy and physically active senior citizens (age: 78.5 ± 5.8 years, height: 163 ± 11.9 cm, and mass: 72.2 ± 19.2 kg; f: n = 24, age: 77.6 ± 5.77 years, height: 157 ± 6.06 cm, and mass: 63.7 ± 11.0 kg; m: n = 27, age: 79.3 ± 5.81 years, height: 168 ± 13.4 cm, and mass: 79.1 ± 21.8 kg) were recruited through advertisements for the research project in local newspapers. They were included if they were at least 70 years of age and were still able to walk without assistance and manage daily tasks without external support. Participants were excluded if they suffered from neurological diseases (such as Alzheimer’s, stroke, or Parkinson’s disease) or orthopedic conditions (abnormal foot deformities or leg length differences) that might influence gait or balance. Further exclusion criteria included artificial joints, prostheses, or metal implants in the lower limbs. Finally, the participants were required to be injury-free in their lower limbs for at least 6 months and present no elevated risk for cardiovascular complications before the examination. These inclusion and exclusion criteria were assessed in a telephone interview.

The participants provided written informed consent, and ethical approval was obtained from the local Ethical Commission (protocol number: FSV 20/038).

### 2.2 Testing procedures

Each individual completed a series of tests in the same order in a single session. First, standing body height and weight were measured using a stadiometer and the InBody 720 System (JP Global Markets GmbH, Eschborn, Germany). Additionally, the foot lengths (heel-to-toe length) of both feet were measured using a specially constructed caliper. The testing protocol comprised normal and maximal walking speed, different static standing tasks, and the STS and TUG tests as common balance parameters (Lohmann et al., 2024).

#### 2.2.1 Gait

Recent literature has proposed the inclusion of gait speed as a viable parameter that should be assessed in comprehensive evaluations for older adults (Beck Jepsen et al., 2022). The normal walking velocity was measured by asking the participants to walk 6 m forward in a straight line over firm ground at their usual (preferred) walking pace used during daily life. All the trials used for data analysis were timed with a stopwatch. For the maximum walking speed, the participants walked the same distance as fast as possible for another two trials (Mehmet et al., 2020).

#### 2.2.2 Timed up and go test

Dynamic balance was assessed using the TUG (Podsiadlo and Richardson, 1991). The participants were instructed to stand up from a chair, then walk 3 m toward a cone as fast as possible, walk around the cone, walk back, and sit on the chair again. Time was measured by the same investigator with a stopwatch. Acceptable reliability and validity of the stopwatch-measured TUG test have been demonstrated previously (Lee et al., 2016; Podsiadlo and Richardson, 1991). Every participant performed a test trial and two timed trials afterward, with the fastest time chosen for data analysis.

#### 2.2.3 Sit-to-stand test

Additionally, the STS was performed as a typical lower-limb strength test in older adults (Cruz-Montecinos et al., 2024; Lord et al., 2002) to simultaneously evaluate balance and postural control (Albalwi and Alharbi, 2023). The five- and ten-times STS were performed, in which participants were instructed to stand up from a chair (45 cm in height) five or ten times as quickly as possible without pushing off the chair. The test was conducted with the arms crossed in front of the chest, and the participants’ starting position was standardized by sitting comfortably with the back against the back of the chair. The trials were classified as valid when the knee and hip joints were fully extended and a safe upright stance was reached (Albalwi and Alharbi, 2023).

#### 2.2.4 Standing tasks

Static balance tests were included to analyze the ability to stabilize the body’s center of mass over the base of support within a given time frame without failure. To account for different underlying strategies to maintain balance at changing base conditions and for test specificity (Batko-Szwaczka et al., 2020), static balance was measured by assessing the stance time and postural sway (center of pressure path length) with a Kistler force plate (Kistler Instrumente GmbH, Sindelfingen, Germany) during three different standing tasks. Participants were instructed to stand as still as possible for 30 s with their hands on their hips and looking straight forward during three different stances of increasing challenge [two-leg, tandem, and one-leg standing (left and right)] (Beck Jepsen et al., 2022; Omaña et al., 2021). For the one-leg stance, the non-standing leg had to be free-hanging without being mounted to the standing leg. The standing time was recorded and analyzed along with the simultaneous sway path.

#### 2.2.5 Lower-limb strength evaluation

Plantar flexor and dorsiflexor strength were assessed with the isokinetic dynamometer (IsoMed 2000, Sindelfingen, Germany). First, the participants were instructed to sit in the dynamometer with one foot for unilateral strength. The testing foot was placed on the platform of the adapter that is designed for the strength assessment of ankle plantar flexion and dorsiflexion. The measurement platform used for ankle strength testing was individually adjusted based on the participant’s shank length. Next, the pivot point of the ankle joint (malleolus lateralis) and the device were synchronized with each other. After that, the seat position was adjusted to the femur length and pelvic anthropometrics so that the knee joint was fixed in a flexed 90° position, while the foot was still positioned on the adapter. With this arrangement, the hip of the tested side was flexed at approximately 45°, while the other side was at 90°. An additional support pole was set at the back of the tested leg to prevent the subject from pushing via leg extension onto the foot platform and thereby falsifying the plantar flexion strength results. Finally, to prevent evasion movements, the foot, ankle, and femur were fixed with straps, the pelvis was strapped with a belt, and the shoulders were restrained with shoulder pads. The participants performed maximal voluntary concentric contractions in the range of −20°–35°, with 0° as the neutral position. The participants were instructed to perform three trials per leg with 2 min of rest in between to avoid fatigue. The results were presented in newton meters and were divided by body weight for further analysis (Stotz et al., 2023b).

### 2.3 Statistical analysis

Data were analyzed using JAMOVI, and the descriptive parameters are provided as the means (M) and standard deviations (SD), while normal distribution was tested using the Shapiro–Wilk test. To account for potential sex-specific relationships, an overall statistic was supplemented by sex-specific subgroup analysis. In case of normal distribution violation, Spearman rank correlations (r_s_) were calculated to explore the relationship between maximal plantar flexion and dorsiflexion strength and walking velocity and the TUG as the primary outcomes. Furthermore, the results of the secondary outcomes for static/dynamic balance, foot length, and the STS were reported. To quantify sex-specific differences between correlations, bivariate Pearson correlation coefficients (r_p_) were additionally calculated and provided with their respective confidence intervals. To determine significant differences between sexes, r_p_ values were z-transformed according to the Fisher method (secondary outcomes). The difference between the two transformed values after standardization was assessed for significance (Diedenhofen and Musch, 2015) (secondary outcomes). Additionally, due to the smaller sample size per subgroup, 95% CIs were considered for interpretation (interpretational restrictions are discussed in the Limitations section). The effect size of the correlation coefficients was determined according to the suggestions of Hopkins (Gogan et al., 2016; Hopkins, 2000) and was defined as trivial if <0.1, small if r ≥ 0.1–< 0.3, moderate if ≥ 0.3–< 0.5, large if ≥ 0.5–< 0.7, very large if ≥ 0.7–< 0.9, and nearly perfect to perfect if ≥0.9. The level of significance was set as α = 0.05.

## 3 Results

**FIGURE 1:**
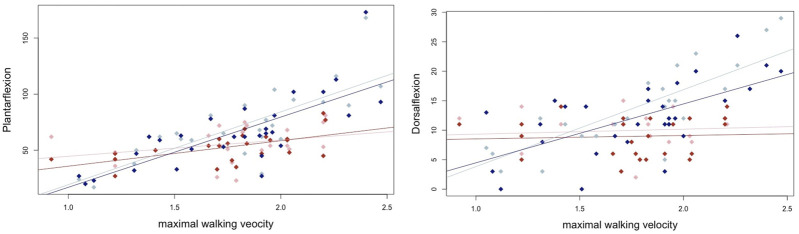
Graphical illustration of the male and female correlations for maximal strength in the plantar flexors and dorsiflexors and the maximal walking velocity, where the blue dots represent male participants and the red dots represent female participants.

**FIGURE 2:**
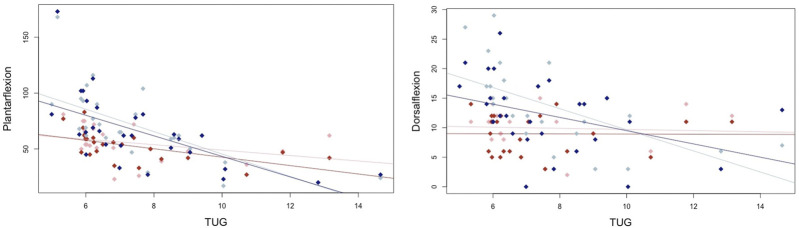
Graphical illustration of the male and female correlations for maximal strength in the plantar flexors and dorsiflexors and the timed up and go test, where the blue dots represent male participants and the red dots represent female participants.

All the participants completed the tests successfully. Normal distribution was present for gait velocity (normal and maximal), tandem stance, maximal dorsiflexion strength in the right leg, and foot lengths, with *p* > 0.05. The other parameters showed *p* < 0.05 and cannot be assumed to be normally distributed. Descriptive statistics are provided in Table 1. The overall and sex-specific subgroup analyses with the individual correlations (r_p_ with 95% CIs and r_s_) and *p*-values are provided in Table 2. Correlations between plantar flexor maximal strength and maximal walking velocity, static balance, and dynamic balance using the TUG test are shown under consideration of sex-specific differences in Figures 1, 2.

**TABLE 1:** Descriptive overall statistics, along with the male- and female-only groups, including all parameters and the test of normal distribution with, values *p* < 0.05 indicating non-normal distribution.

Parameter	M ± SD	Median	25–75 percentile	Minimum–maximum	N	Shapiro–Wilk (*p*)
Overall						
Normal velocity (in m/s)	1.33 ± 0.28	1.35	1.15–1.51	0.69–1.85	51	0.16
Maximum velocity (in m/s)	1.76 ± 0.36	1.83	1.53–1.97	0.92–2.47	51	0.18
Two-leg stance (CoP) (in mm)	28.0 ± 14.3	22.6	18.8–35.3	12.3–71.0	51	<0.001
Semi-tandem (CoP) (in mm)	41.0 ± 21.0	33.8	24.5–52.1	15.9–98.7	51	<0.001
Tandem (CoP) (in mm)	68.0 ± 24.4	61.3	50.0–83.3	26.1–133	39	0.17
Time tandem (in s)	26.6 ± 8.52	30	30.0–30.0	0–30	47	0.17
Time one leg (L) (in s)	14.0 ± 13.6	10	0.0–30.0	0–30	51	<0.001
Time one leg (r) (in s)	14.1 ± 13.4	11	0.0–30.0	0–30	51	<0.001
TUG (in s)	7.47 ± 2.10	6.81	6.01–8.22	5.0–14.7	51	<0.001
STS-5 (in s)	8.78 ± 3.06	8.26	6.89–9.30	5.24–24.1	51	<0.001
STS-10 (in s)	18.0 ± 4.74	17.2	14.7–19.3	10.9–34.1	50	0.002
F_max_ PF (L) (in Nm)	63.1 ± 26.2	62	50.0–72.0	17–168	51	0.001
F_max_ PF (r) (in Nm)	59.7 ± 25.9	59	45.0–66.0	20–173	51	0.03
F_max_ DF (L) (in Nm)	12.1 ± 5.6	12	9.0–15.0	2–29	51	<0.001
F_max_ DF (r) (in Nm)	10.7 ± 5.29	11	6.0–14.0	0–26	51	0.35
Foot length (L) (in cm)	25.2 ± 1.93	25.3	23.9–27.0	20.0–28.5	51	0.25
Foot length (r) (in cm)	25.3 ± 1.80	25.5	24.0–26.5	20.4–28.5	51	0.54
Male participants						
Normal velocity (in m/s)	1.28 ± 0.29	1.28	1.1–1.5	0.71–1.85	27	0.90
Maximum velocity (in m/s)	1.77 ± 0.39	1.83	1.5–2.0	0.97–2.47	27	0.51
Two-leg stance(CoP) (in mm)	32.6 ± 15.8	27.4	21.0–39.1	14.7–71.0	27	0.004
Semi-tandem(CoP) (in mm)	46.4 ± 23.9	42.0	25.1–60.0	17.3–98.7	27	0.033
Tandem (CoP) (in mm)	76.2 ± 20.7	80.2	55.3–95.4	45.3–110	20	0.15
Time tandem (in s)	25.6 ± 10.1	30	30.0–30.0	0–30	25	<0.001
Time one leg (L) (in s)	10.9 ± 13.1	—	0.0–30.0	0–30	27	<0.001
Time one leg (r) (in s)	10.1 ± 12.4	5	0.0–30.0	0–30	27	<0.001
TUG (in s)	7.62 ± 2.20	7.03	6.0–8.6	5.0–14.7	27	<0.001
STS-5 (in s)	9.37 ± 3.85	8.29	6.8–11.0	5.53–24.1	27	<0.001
STS-10 (in s)	18.7 ± 5.68	17.1	14.6–22.5	10.9–34.1	27	0.05
F_max_ PF (L) (in Nm)	69.4 ± 31.7	65	59.0–90.0	17–168	27	0.08
F_max_ PF (r) (in Nm)	65.3 ± 32.0	62	47.0–81.0	20–173	27	0.01
F_max_ DF (L) (in Nm)	13.9 ± 6.43	14	11.0–17.0	3–29	27	0.56
F_max_ DF (r) (in Nm)	12.2 ± 6.18	12	9.0–15.0	0–26	27	0.79
Foot length (L) (in cm)	26.5 ± 1.42	26.9	25.5–27.5	23.2–28.5	27	0.155
Foot length (r) (in cm)	26.5 ± 1.28	26.5	25.6–27.4	25.5–28.5	27	0.480
Female participants						
Normal velocity (in m/s)	1.38 ± 0.25	1.40	1.33–1.5	0.69–1.73	24	0.03
Maximum velocity (in m/s)	1.75 ± 0.34	1.81	1.69–2.0	0.92–2.21	24	0.04
Two-leg stance (CoP) (in mm)	22.5 ± 10.3	20.3	16.0–23.6	12.3–53.0	24	<0.001
Semi-tandem (CoP) (in mm)	35 ± 15.6	31.5	24.0–41.3	15.9–79.3	24	0.02
Tandem (CoP) (in mm)	59.4 ± 25.6	53.7	30.1–61.1	26.1–133	19	0.01
Time tandem (in s)	27.8 ± 6.22	30	30.0–30.0	5–30	22	<0.001
Time one leg (L) (in s)	17.8 ± 13.4	24	2.25–30.0	0–30	24	<0.001
Time one leg (r) (in s)	19.0 ± 13.2	27.5	3.75–30.0	0–30	24	<0.001
TUG (in s)	7.29 ± 2.00	6.41	5.98–7.64	5.34–13.2	24	<0.001
STS-5 (in s)	8.05 ± 1.44	7.90	7.08–8.94	5.24–11.8	24	0.64
STS-10 (in s)	17.1 ± 3.23	17.2	15.0–18.9	11.2–26.2	24	0.48
F_max_ PF (L) (in Nm)	55.5 ± 15.0	54.0	49.5–66.5	23–81	24	0.43
F_max_ PF (r) (in Nm)	52.9 ± 13.3	54	44.3–60.0	27–83	24	0.94
F_max_ DF (L) (in Nm)	9.96 ± 3.44	11	8.0–12.0	2–15	24	0.12
F_max_ DF (r) (in Nm)	8.96 ± 3.28	10	6.0–11.3	3–14	24	0.03
Foot length (L) (in cm)	23.8 ± 1.34	23.8	23.1–24.6	20–26	24	0.45
Foot length (r) (in cm)	23.9 ± 1.29	23.9	23.4–24.9	20.4–26.0	24	0.42

DF, dorsiflexion; F_max_, maximal force; PF, plantar flexion; STS, sit-to-stand; TUG, timed up and go; CoP, center of pressure.

**TABLE 2:** Correlation coefficients are provided as overall statistics for the relationship between maximal plantar flexion and dorsiflexion strength and the gait and balance parameters.

Parameter	Plantar flexion L (r_p_) with 95% CI	Plantar flexion R (r_p_) with 95% CI	Dorsiflexion L (r_p_) with 95% CI	Dorsiflexion R (r_p_) with 95% CI	Plantar flexion L (r_s_)	Plantar flexion R (r_s_)	Dorsiflexion L (r_s_)	Dorsiflexion R (r_s_)
V_norm_	**0.50**; 0.27–0.68 (<0.001)*	**0.54**; 0.31–0.71 (<0.001)*	**0.37**; 0.11–0.58 (0.007)*	**0.34**; 0.08–0.56 (0.013)*	0.51 (<0.001)*	0.51 (<0.001)*	0.31 (0.023)*	0.28 (0.045)*
V_max_	**0.64**; 0.44–0.77 (<0.001)*	**0.67**; 0.49–0.80 (<0.001)*	**0.55**; 0.33–0.71 (<0.001)*	**0.45**; 0.20–0.64 (<0.001)*	0.62 (<0.001)*	0.66 (<0.001)*	0.51 (<0.001)*	0.42 (0.002)*
Two-leg stance CoP sway	−0.02; −0.29–0.25 (0.886)	−0.02; −0.29–0.25 (0.883)	0.07; −0.21–0.33 (0.645)	0.048; −0.23–0.32 (0.733)	−0.002 (0.991)	−0.005 (0.971)	0.112 (0.423)	0.023 (0.868)
Semi-tandem CoP sway	0.015; −0.26–0.29 (0.916)	0.003;-0.28–0.29 (0.983)	−0.015; −0.29–0.26 (0.92)	0.057; −0.22–0.33 (0.690)	−0.093 (0.513)	−0.089 (0.532)	0.00 (0.998)	0.023 (0.873)
Tandem CoP sway	−0.002; −0.32–0.31 (0.992)	0.041; −0.28–0.35 (0.802)	0.04; −0.28–0.35 (0.817)	0.078; −0.24–0.38 (0.635)	−0.21 (0.19)	−0.10 (0.536)	0.042 (0.801)	0.10 (0.566)
Tandem time	0.17; −0.13–0.43 (0.267)	0.20: 0.09–0.46 (0.177)	0.18; −0.12–0.44 (0.177)	0.14; −0.16–0.41 (0.359)	0.24 (0.111)	0.30 (0.043)*	0.23 (0.124)	0.15 (0.327)
One-leg time, left	0.36; 0.1–0.57 (0.008)	0.35; 0.09–0.57 (0.011)	0.28; 0.09–0.51 (0.044)	0.20; −0.07–0.45 (0.149)	0.43 (0.001)*	0.45 (<0.001)*	0.22 (0.11)	0.19 (0.185)
One-leg time, right	0.31; 0.04–0.53 (0.026)	0.35; 0.09–0.57 (0.01)	0.20; −0.07–0.45 (0.15)	0.20; −0.07–0.45 (0.15)	0.31 (0.002)*	0.50 (<0.001)*	0.19 (0.168)	0.23 (0.101)
TUG	−0.53; −0.70 to −0.31 (<0.001)	−0.55; −0.71 to −0.32 (<0.001)	−0.38; −0.59 to −0.13 (0.005)	−0.24; −0.48–0.03 (0.08)	−0.53 (<0.001)*	−0.64 (<0.001)*	−0.45 (<0.001)*	−0.31 (0.025)*
STS-5	−0.44; −0.19 to −0.64 (0.001)	−0.40; −061 to −0.14 (0.004)	−0.29; −0.53 to −0.02 (0.036)	−0.28; −0.52 to −0.007 (0.045)	−0.49 (<0.001)*	−0.44 (0.001)*	−0.27 (0.056)	−0.23 (0.098)
STS-10	−0.42; −0.63 to −0.16 (0.002)	−0.37; −0.59 to −0.10 (0.008)	−0.27; −0.51–0.005 (0.055)	−0.20; −0.45–0.09 (0.173)	−0.50 (<0.001)*	−0.44 (0.002)*	−0.25 (0.083)	−0.19 (0.180)
Foot length, left	**0.33**; 0.07–0.55 (0.015)*	**0.30**; 0.04–0.53 (0.028)*	**0.40**; 0.14–0.60 (0.003)*	**0.44**; 0.20–0.64 (<0.001)*	0.25 (0.069)	0.24 (0.087)	0.33 (0.016)*	0.42 (0.002)*
Foot length, right	**0.34**; 0.08–0.56 (0.012)*	**0.32**; 0.05–0.54 (0.021)*	**0.40**; 0.15–0.54 (0.003)*	**0.44**; 0.19–0.64 (<0.001)*	0.25 (0.071)	0.23 (0.092)	0.32 (0.02)*	0.41 (0.003)*
Male participants								
V_norm_	**0.74**; 0.0.50–0.87 (<0.001)*	**0.69**; 0.43–0.84 (<0.001)	**0.67**; 0.43–0.84 (<0.001)	**0.60**; 0.30–0.79 (<0.001)	0.79 (<0.001)*	0.75 (<0.001)*	0.74 (<0.001)*	**0.60 (<0.001)**
V_max_	**0.80**; 0.61–0.90 (<0.001)	**0.76**; 0.54–0.88 (<0.001)	**0.79**; 0.59–0.90 (<0.001)	**0.63**; 0.34–0.81 (<0.001)	0.79 (<0.001)*	0.79 (<0.001)*	0.77 (<0.001)*	0.61 (<0.001)*
Two-leg stance CoP sway	−0.13; −0.47–0.25 (0.51)	−0.12; −0.47–0.26 (0.53)	−0.20; −0.53–0.18 (0.30)	−0.22; −0.54–0.16 (0.25)	−0.11 (0.572)	−0.05 (0.818)	−0.09 (0.668)	0.001 (0.996)
Semi-tandem CoP sway	−0.09; −0.45–0.29 (0.65)	−0.08;-0.44–0.30 (0.68)	−0.23; −0.55–0.16 (0.25)	−0.19; −0.53–0.19 (0.33)	0.03 (0.885)	0.06 (0.770)	−0.01 (0.95)	0.09 (0.650)
Tandem CoP sway	−0.22; −0.61–0.24 (0.35)	−0.044; −0.48–0.41 (0.85)	−0.28; −0.64–0.19 (0.23)	−0.13; −0.54–0.34 (0.60)	−0.43 (0.058)	−0.25 (0.292)	−0.22 (0.363)	−0.09 (0.709)
Tandem time	0.20; −0.21–0.55 (0.33)	0.24: 0.18–0.58 (0.26)	0.19; −0.22–0.55 (0.35)	0.21; −0.20–0.56 (0.31)	0.31 (0.129)	0.37 (0.072)	0.33 (0.110)	0.27 (0.190)
One-leg time, left	**0.47**; 0.12–0.71 (0.01)	**0.42**; 0.06–0.68 (0.02)	**0.51**; 0.18–0.74 (0.005)	**0.38**; 0.02–0.66 (0.04)	0.58 (0.001)*	0.55 (0.002)*	0.52 (0.004)*	0.40 (0.033)*
One-leg time, right	**0.51**; 0.17–0.74 (0.005)	**0.51**; 0.16–0.74 (0.005)	**0.51**; 0.18–0.74 (0.005)	**0.52**; 0.19–0.75 (0.004)	0.62 (<0.001)*	0.66 (<0.001)*	0.53 (0.003)*	0.55 (0.002)*
TUG	**−0.70**; −0.85 to −0.45 (<0.001)	**−0.63**; −0.81 to −0.35 (<0.001)	**−0.62**; −0.80 to −0.32 (<0.001)	**−0.40**; −0.67 to −0.04 (0.03)	−0.77 (<0.001)*	**−0.72 (<0.001)**	−0.70 (<0.001)*	−0.49 (0.008)*
STS-5	**−0.59**; −0.28 to −0.79 (<0.001)	**−0.51**; −0.74 to −0.17 (0.006)	**−0.49**; −0.73 to −0.15 (0.008)	**−0.43**; −0.69 to −0.07 (0.022)	−0.67 (<0.001)*	−0.57 (0.002)*	−0.45 (0.018)*	−0.32 (0.009)*
STS-10	**−0.57**; −0.78 to −0.24 (0.002)	**−0.48**; −0.73 to −0.12 (0.012)	**−0.49**; −0.73 to −0.13 (0.01)	**−0.34**; −0.64–0.04 (0.08)	−0.65 (<0.001)*	−0.54 (0.004)*	−0.41 (0.034)*	−0.30 (0.134)
Foot length, left	0.16; −0.22–0.50 (0.41)	0.21; −0.17–0.54 (0.28)	0.27; −0.11–0.58 (0.15)	**0.42**; 0.07–0.68 (0.02)	0.09 (0.649)	0.19 (0.327)	0.26 (0.174)	0.46 (0.013)*
Foot length, right	0.20; −0.18–0.53 (0.29)	0.25; −0.13–0.56 (0.19)	0.30; −0.08–0.60 (0.12)	**0.45**; 0.11–0.70 (0.013)	0.15 (0.451)	0.22 (0.258)	0.29 (0.132)	0.50 (0.005)*
Female participants								
V_norm_	0.19; −0.24–0.55 (0.38)	**0.46**; 0.07–0.73 (0.02)	−0.07; −0.46–0.34 (0.74)	−0.03; −0.43–0.38 (0.90)	0.32 (0.134)	0.37 (0.076)	−0.18 (0.389)	−0.02 (0.922)
V_max_	0.33; −0.09–0.65 (0.12)	**0.58**; 0.23–0.80 (0.003)	0.08; −0.33–0.47 (0.70)	0.06; −0.35–0.45 (0.79)	0.46 (0.025)*	0.56 (0.004)*	0.14 (0.520)	0.21 (0.334)
Two-leg stance CoP sway	−0.09; −0.48–0.33 (0.68)	−0.26; −0.60–0.16 (0.23)	0.17; −0.25–0.54 (0.43)	0.043; −0.37–0.44 (0.84)	−0.17 (0.437)	−0.27 (0.203)	−0.044 (0.838)	−0.30 (0.156)
Semi-tandem CoP sway	−0.20; −0.56–0.22 (0.36)	**−0.46**;-0.73 to −0.07 (0.02)	−0.06; −0.45–0.36 (0.80)	−0.08; −0.47–0.33 (0.70)	−0.402 (0.051)	−0.55 (0.005)*	−0.11 (0.602)	−0.23 (0.286)
Tandem CoP sway	−0.13; −0.55–0.35 (0.60)	−0.28; −0.65–0.21 (0.26)	0.02; −0.44–0.47 (0.92)	−0.06; −0.50–0.41 (0.81)	0.04 (0.849)	0.14 (0.519)	−0.04 (0.84)	−0.06 (0.768)
Tandem time	0.35; −0.09–0.67 (0.35)	0.39; −0.04–0.69 (0.08)	**0.51**; 0.11–0.77 (0.02)	0.23; −0.22–0.59 (0.31)	0.34 (0.123)	0.41 (0.059)	0.36 (0.009)	0.20 (0.365)
One-leg time, left	**0.53**; 0.17–0.77 (0.007)	**0.63**; 0.30–0.82 (0.001)	0.24; −0.18–0.59 (0.25)	0.18; −0.24–0.54 (0.40)	0.56 (0.005)*	0.71 (<0.001)*	0.12 (0.577)	0.26 (0.218)
One-leg time, right	0.36; −0.05–0.67 (0.09)	**0.53**; 0.16–0.77 (0.008)	0.10; −0.32–0.49 (0.64)	−0.005; −0.41–0.40 (0.98)	0.55 (0.005)*	0.72 (<0.001)*	0.12 (0.583)	0.15 (0.476)
TUG	−0.33; −0.65–0.09 (0.12)	**−0.57**; −0.79 to −0.22 (0.004)	−0.06; −0.41–0.36 (0.77)	−0.01; −0.41–0.40 (0.97)	−0.59 (0.002)*	**−0.72 (<0.001)**	−0.22 (0.299)	−0.16 (0.450)
STS-5	−0.24; 0.19 to −0.60 (0.27)	−0.27; −0.62–0.16 (0.21)	0.02; −0.40–0.43 (0.94)	−0.05; −0.45–0.37 (0.84)	−0.28 (0.192)	−0.29 (0.179)	−0.05 (0.807)	−0.05 (0.833)
STS-10	**−0.42**; −0.63 to −0.16 (0.002)	**−0.37**; −0.59 to −0.10 (0.008)	−0.27; −0.51–0.005 (0.055)	−0.20; −0.45–0.09 (0.173)	−0.36 (0.089)	−0.37 (0.084)	−0.05 (0.815)	−0.02 (0.924)
Foot length, left	0.39; −0.02–0.68 (0.06)	0.19; −0.23–0.55 (0.37)	0.14; −0.28–0.52 (0.50)	0.17; −0.25–0.54 (0.43)	0.23 (0.279)	0.06 (0.781)	−0.06 (0.797)	0.10 (0.632)
Foot length, right	0.33; −0.08–0.65 (0.11)	0.18; −0.25–0.54 (0.41)	0.10; −0.31–0.49 (0.64)	0.11; −0.31–0.49 (0.61)	0.19 (0.367)	0.04 (0.866)	−0.09 (0.661)	0.028 (0.896)

CoP, center of pressure, r_p_, Pearson Correlation, r_s_, Spearman Correlation, STS, sit to stand; TUG, timed up and go test, 95% CI, 95% confidence intervals.

Bold underlines that the effect size reached the level of significance (p < 0.05).

*significant correlation.

### 3.1 Overall results

Due to the violation of the normal distribution assumption, r_s_ values were calculated, showing 25 significant correlations with small to large magnitude effects in the overall sample. Maximal strength in plantar flexion was correlated with normal walking velocity (r_s_ = 0.51, p < 0.001) and maximal walking velocity (r_s_ = 0.62–0.66, *p* < 0.001). Dorsiflexion strength showed small to moderate relationships (r_s_ = 0.28–0.51, *p* < 0.001–0.045). Single-leg stance time (for the right leg) and tandem stance time showed moderate correlations (r_s_ = 0.30–0.50, *p* < 0.001–0.043). Similar (negative) relationships were found for dynamic balance and complex tests (TUG and STS) (r_s_ = −0.44 to −0.64, *p* < 0.001–0.002) in plantar flexion strength, while dorsiflexion strength showed no effects. The progressively increasing task complexity in the standing tasks is expressed in a reduced success rate for the tandem stance, with 12 participants unable to complete the entire task and, therefore, excluded from the CoP evaluation. In addition, one participant was not able to complete the STS10 (see Table 1).

### 3.2 Correlations in male participants

In the male-only subgroup, there were 29 significant correlations reaching moderate to very large effect size magnitudes (up to r_s_ = 0.79). Plantar flexion maximum strength showed correlations with r_s_ = 0.75–0.79 for normal walking velocity and r_s_ = 0.79 (*p* < 0.001) for maximal walking velocity. Likewise, dorsiflexion strength was correlated with walking velocities, with r_s_ = 0.60–0.77, indicating an increased relationship compared to that of the overall sample. The one-leg stance time showed, again, positive relationships with strength (r_s_ = 0.40–0.62, *p* < 0.001–0.03), while the TUG and STS parameters were again significantly correlated with plantar flexion strength (r_s_ = −0.54–0.67), and one dorsiflexion correlation (STS-10, right leg) did not reach the level of significance (r_s_ = −0.30, *p* = 0.134). For further and more detailed results, including 95% CIs, of r_p_, see Table 2.

### 3.3 Correlations in female participants

Nine significant correlations, ranging from small to large magnitude effects, were found in the female subgroup. Normal walking velocity was not correlated anymore, while V_max_ showed correlations between r_s_ = 0.46–0.56 (*p* = 0.004–0.025) for plantar flexion strength. Dorsiflexion strength showed no relationships (*p* = 0.334–0.922). Semi-tandem stance CoP showed a significant correlation for the right leg plantar flexion strength (r_s_ = 0.55, *p* = 0.005). One-leg stance time was again correlated with the maximum strength in the plantar flexion (r_s_ = 0.55–0.71, *p* < 0.001–0.005) without reaching the level of significance for dorsiflexion strength. While in the overall results and the male subgroup, there were significant correlations of the STS with strength, these were not observed in female participants. Only the TUG test was correlated with the plantar flexion strength in both legs (r_s_ = −0.59 to −0.72, *p* < 0.001–0.002).

### 3.4 Sex-specific difference in correlations

Pearson correlations and Spearman correlation coefficients resulted in slightly different classifications. While r_p_ indicated a significant correlation of the maximal dorsiflexion strength on STS-10 in the right leg, r_s_ became non-significant in male participants. In female participants, while r_p_ indicated a significant influence of right maximal plantar flexion strength on normal walking speed, r_s_ changed to *p* = 0.076. Similarly, two correlations on STS-10 reduced the certainty and slightly failed to reach the level of significance for r_s_ (*p* = 0.089, *p* = 0.084) compared to r_p_ (*p* = 0.002 and *p* = 0.008). On the other hand, two insignificant r_p_ values reached the level of significance (one-leg stance and TUG) when using r_s_ (r_s_ = 0.55 and −0.59, *p* = 0.005 and *p* = 0.002). For detailed comparisons, please see Table 2.

There were 30 significant correlations (r_p_) in the male-only group but only nine in the female only group. Nevertheless, considering z-transformed data to stratify correlations for sex showed significant male–female differences in only a few cases. There were significant differences between plantar flexor and dorsiflexor strength and mean velocity (*p* = 0.003–0.011), with male participants showing r_p_ = 0.74 (0.50–0.87 95% CI) in the left leg, while female correlations did not reach the level of significance (r_p_ = 0.19, −0.24–0.55 95% CI). Such a difference was only indicated in the left leg, while a non-significant difference was detected in the right plantar flexor strength (see Table 2 for r_p_ and 95% CI). Similarly, for maximal velocity, male participants reached an effect size of r_p_ = 0.80 and 0.61–0.90 95% CI (*p* < 0.001), while female participants reached r_p_ = 0.33 and −0.09 – 0.65 95% CI (*p* = 0.68) in the left leg. Similarly, male participants showed significant dorsiflexion strength influence on TUG performance (r_p_ = −0.62, −0.80 to −0.32 95% CI, r_p_ = −0.40, −0.67 to −0.04, 95% CI for the right and left leg, respectively (see Table 2)). In accordance with the inference statistics test after Fisher z-transformation indicating a significant sex-specific difference (*p* = 0.001–0.023), in female participants, no significant effects were found for dorsiflexion strength on TUG performance (r_p_ = −0.06 to −0.01, −0.41 – 0.40 95% CI, *p* = 0.77–0.97). Nevertheless, the overlap of 95% CIs reduces the certainty of significance for these differences. For the remaining correlations, no sex-related differences between strength and the parameters of static or dynamic balance or the STS tests could be detected.

## 4 Discussion

The study was designed to analyze the influence of lower-extremity strength on different balance and walking parameters that are often considered important in fall prevention routines in older adults. While previous study results highlighted the relevance of strength capacity in, for instance, hip muscles (Hicks et al., 2012) or leg extensors (Fukagawa et al., 1995; Stotz et al., 2023a), on balance or walking, limited attention has been paid to ankle joint muscle strength. This limitation is mirrored in the current rather superficial strength training recommendations for older adults, which often fail to provide specific exercises for specific purposes, such as balance or gait improvements (McDermott and Mernitz, 2006). Although correlations do not prove a causal relationship between strength capacity and balance in the evaluated population, this study provides the first indications for future specialized studies. More specifically, this study showed that lower-limb strength had a moderate to large relationship with relevant gait and balance parameters, such as walking velocity, one-leg stance, and combined tasks such as the TUG and STS. Showing even larger effects compared to previous studies (Tavakkoli Oskouei et al., 2021) in male participants, the presented results do not exclusively indicate a stronger consideration of plantar flexion and dorsiflexion strength in future longitudinal studies to analyze the causal relationship but also call for further investigations on sex-specific exercise and training recommendations (Chung et al., 2023).

### 4.1 Walking speed

A decline in walking speed is associated with several age-related clinical conditions, such as an increased risk of falls and hospitalization and physical and cognitive impairments (Mielke et al., 2013). It is, therefore, not surprising that considerable attention has been focused on identifying factors that can improve walking speed in older adults. In our study, the normal and maximal walking speed correlation coefficients showed moderate to large positive relationships, with an emphasis on male participants. Female participants showed partially non-significant or small-to-large magnitude relationships with normal walking velocity (r_s_ = −0.02 – 0.37) and maximal (r_s_ = 0.14–0.56) walking velocity. Although the highest effect size was found for right plantar flexion strength on the maximal walking velocity (rs = 0.56, *p* = 0.004), in accordance with the male correlations, these showed superior effect sizes with r_s_ = 0.79, *p* < 0.001. The same phenomenon can be observed in the TUG test, which can be considered a combination of balance and walking speed (Podsiadlo and Richardson, 1991). Accordingly, previous literature outlined the relevance of combined knee extension, knee flexion, and ankle dorsiflexion strength when testing the 6-m walking speed (Tiedemann et al., 2005).

Even though correlations do not prove a causal relationship between lower-leg strength capacity in the elderly population and the ability to walk at high velocities, the results could indicate a certain importance of plantar flexion and dorsiflexion strength for gait motor control. Although these results agree with previous literature (Drey et al., 2012; Zech et al., 2012) and call for future studies on the potential of ankle muscle strength training for gait, the comparatively large 95% confidence intervals indicate that either further individual factors influence this relationship or that the relationship is unstable, which necessitates studies with larger sample sizes. Accordingly, Schönbrodt and Perugini (2013) indicated that stable correlation estimates required a sample size of approximately n = 250 in typical scenarios. Although such a large sample size was not present in this study, comparable analyses in the literature have included even smaller sample sizes. This caveat also applied to maximal walking gait velocities when comparing correlations between female and male participants. Male correlations were higher, with correlations up to r_s_ = 0.79, while in female participants, the highest relationship was observed for right plantar flexor strength and maximal walking velocity with r_s_ = 0.56. Although the z-transformed inference statistics comparison indicated significant differences between male and female participants for maximal walking velocity (and for mean velocity and the TUG), 95% CIs still overlapped between the subgroup samples. This could possibly be attributed to the wide range of 95% CIs caused by the small sample size.

Previous studies have already shown higher strength values in male participants than in female participants (Gao et al., 2025; Lu et al., 2020; Warneke et al., 2023b). Therefore, it could be speculated that strength was a determining factor for maximal walking velocity, potentially resulting in higher walking velocities with longer stride lengths in male participants (Houle et al., 2025). This hypothesis, however, remains speculative in this study as the stride length was not measured, and (again) correlations did not provide a causal relationship. Nevertheless, higher maximal strength was related to higher walking velocities, with a stronger relationship observed in male participants (even with underpowered subgroup sampling). Accordingly, future studies should evaluate stride length (e.g., via 3D motion capture and force plate analysis) and analyze whether an increase in maximal plantar flexor strength increases the relevant gait parameters.

Potential sex-specific differences require further research in order to better understand the contributing factors and analyze the potential of different exercise interventions for men and women. Nonetheless, the positive influence of maximal lower-leg strength on walking velocity appears logical from both a physiological and functional perspective. While walking (and running or other such activities), velocity benefit from absorbing and generating high forces to the ground to produce impulses for moving forward (Novacheck, 1998), and considering that action = reaction, the strength capacity might become a pronounced performance predictor. Accordingly, previous research supports this relationship in similar populations (Stotz et al., 2023a; Tavakkoli Oskouei et al., 2021). Although the overlap of 95% CIs requires careful interpretation, the indicated potential sex-specific differences indicate that this relationship is more prominent in male participants than in female participants. Research supports sex-specific strategies to enhance walking performance (Baudendistel et al., 2021). While male participants appear to cover distance with longer stride length, thereby enhancing the exerted forces, female participants seem to increase step frequency, while their stride lengths remain less affected (Park et al., 2018). Whether female participants alter this economization strategy in response to improved maximal strength (i.e., stride length enhances in response to/highly correlated with enhanced maximal strength in the plantar flexors) remains a matter for future research and necessitates longitudinal studies that include effective training routines and extended training periods.

### 4.2 Static balance

In contrast to walking speed and dynamic balance (TUG and STS), the CoP sway velocity was not significantly correlated with the strength capacity in the lower leg. This applies to both the simpler standing conditions (two-legged stance) and the challenging conditions (tandem and one-legged stance). Although Muehlbauer et al. (2015) found significant correlations between maximal strength and balance performance, the literature regarding isolated strength in lower-leg muscles is scarce. Findings extracted from a previous meta-analysis (Muehlbauer et al., 2015) are rather outdated and, due to one-dimensional analytical models and intra-study dependencies of multiple outcomes, potentially overestimated. Although there is literature supporting meaningful associations between plantar flexor and dorsiflexor strength and CoP measurements, further studies are required to clarify whether other muscle function variables, such as the rate of force development or the strength steadiness of plantar flexor muscles, play a superior role in static balance than maximum torque production capacity (Kouzaki and Shinohara, 2010). Another aspect hampering the interpretability and generalization of the existing studies is the variability in testing protocols. The validity of muscle strength testing depends on the specific task and test conditions (Warneke et al., 2023a). In addition, balance tests are highly task-specific, and findings from one task cannot be automatically applied to other tasks (Kümmel et al., 2016). Standing postural-control abilities (including sway) appear to be particularly influenced by joint somatosensory information from the lower legs (Fitzpatrick and McCloskey, 1994). Since this also includes information on the muscle spindle, a small relationship between muscle strength abilities and standing postural sway was expected in this study. Considering our observed lack of associations, one may speculate that the neuromuscular and proprioceptive abilities needed for static balance control are not closely related to maximal strength capacities and must be considered distinct constructs. Furthermore, the increasing difficulty from the two-leg stance to the semi-tandem, tandem, and one-leg stance showed no moderation of the muscle strength vs. sway velocity correlation, while stance time during the more challenging standing conditions was significantly associated with plantar flexion strength. It could, therefore, be speculated that both parameters are indicators of general high function in older adults, but muscle strength plays only a minor role in maintaining upright static standing. Previous research has outlined that, especially in fitter older adults, the relevance of maximal strength on the balance tasks decreased, potentially because balance must be considered a multifactorial and complex construct that is influenced by a complex network of different systems, such as the central nervous system, vestibular system, and the visual and proprioceptive systems.

Only the one-leg standing time was slightly correlated with the strength capacity. Since not all participants were able to complete the task, it can be assumed that quiet standing on one leg is the most challenging static balance task for older adults and may require some strength capacities. Therefore, greater lower-leg strength probably helps maintain the one-leg standing posture over a longer period. However, the current study does not distinguish between maximal strength and strength endurance, which appears to be physiologically more related to 30 s standing tasks in older adults than the ability to produce maximal force output.

### 4.3 Sit-to-stand ability

Although frequently mentioned in balance tests for older adults, the STS can also be considered a multifactorial test that includes dynamically lifting the body mass from a chair (Zech et al., 2011) and serves as a measure of muscle strength in older adults (Lord et al., 2002). While mimicking a half-squat movement repeatedly, which could be assumed to be more related to the knee and hip extensors (Corrigan and Bohannon, 2001), the presented results are in accordance with the literature (Lord et al., 2002) as most of the calculated correlations showed moderate and significant associations between plantar flexor and dorsiflexor strength and STS. Since maximal strength capacities are rarely limited to one exclusive muscle group but are an indicator of whole-body muscle strength, it can be speculated that participants with strong quadriceps and glutes also have stronger lower-leg muscles. However, future studies may be necessary to analyze these associations. Previous studies on the relationship between handgrip and knee extension/flexion strength have demonstrated controversial results (Alonso et al., 2018). In our study, participants with higher muscle strength showed an association with strength and power values in the STS test, with up to r_s_ = −0.67 (in male participants).

### 4.4 Timed up-and-go test

The TUG is a common component of health assessments for older adults (Bergquist et al., 2019) and has demonstrated validity for predicting short-term mortality and hospitalization (Chua et al., 2020; 2025). With outlined significant relationships to upper- and lower-limb muscle strength (via STS), balance (one-leg stance), maximal and normal velocity, and aerobic capacity (Coelho-Junior et al., 2018), the TUG serves as an indicator for physical fitness in older adults (Idland et al., 2013) that combines several health-related abilities (Benavent-Caballer et al., 2016). While the TUG is obviously correlated with thigh muscle strength as standing up via the STS (as a component of the TUG) (Kwan et al., 2011), the presented results confirmed a large magnitude of influence of plantar flexor strength (rs = 0.63, *p* < 0.001) in the overall sample size, with a peak in the male subgroup of r_s_ = 0.77, *p* < 0.001 and r_s_ = 0.72, *p* < 0.001 in the female participants. Interestingly, as a combination of standing up and walking a prescribed path as fast as possible, correlations in the STS (plantar flexion strength: r_s_ = 0.54–0.67, *p* < 0.001–0.034) were similar to those of the TUG (r_s_ = −0.49 – 0.72, *p* < 0.001–0.008 for plantar and dorsiflexion strength) in male participants, while in female participants, the STS was not significantly correlated with strength parameters (*p* = 0.084–0.924). Therefore, it could be hypothesized that for TUG, strength had a different function in male participants compared to that in female participants. Whether this discrepancy can be attributed to sex-specific differences or differences in baseline performance that are occasionally observable between male and female participants remains a matter of debate for future research (e.g., performance-matched male versus female comparisons). Nevertheless, the results require further studies on the role of maximal strength in moderating balance and mobility in older adults (Idland et al., 2013).

### 4.5 Outlook

Nevertheless, causal relationships remain to be analyzed via longitudinal testing designs, affecting lower-body muscle strength to explore its effectivity on gait, balance, and functional parameters over long intervention periods.

Taken together, it appears that female participants show a different relationship between lower-leg strength and walking and balance parameters. Higher strength parameters in male participants could be attributed to their greater body weight, which must be accelerated when moving forward. However, assuming a positive relationship between strength and walking and dynamic balance in male participants would justify the demand for longitudinal research; to us, it would be even more interesting to explore whether correlations in female participants would change if strength capacity were enhanced through training.

### 4.6 Limitations

Some limitations of this study must be acknowledged. First, many outcomes were not normally distributed. On the one hand, violating the normal distribution assumption in correlation analysis might only marginally influence the results, especially if the sample size was adequately chosen (which was the case at least in the overall sample). On the other hand, inferential statistics and the corresponding *p*-values should be interpreted with caution. Although in the male subgroup, *post hoc* power analysis for r_p_ = 0.7–0.8 (walking velocity as primary outcomes) showed a power of up to 0.99%, the sample size was not large enough to reach sufficient power (0.13). To counteract this, r_p_ reporting was supplemented by an r_s_ calculation that showed comparatively high robustness even in the (underpowered) subgroup comparisons of the r_p_. Nevertheless, the comparatively small sample of 24 female participants requires future research with larger subgroup sample sizes as the study design was underpowered for subgroup analyses. This limitation is particularly relevant to the one-leg stance test as several participants were unable to perform this task. The inability of many participants to adequately stand on one leg resulted in a markedly high dropout rate, preventing the statistical analysis of the sway area from the one-leg stance tests. Here, in addition to gait velocities, numerous additional parameters could theoretically be calculated. For instance, in balance tasks, the general CoP sway is sometimes reported separately for anterior–posterior sway and medial–lateral sway. Furthermore, parameters such as the convex hull could supplement the presented analysis. In addition, for walking parameters, future studies might include further biomechanical parameters, such as stride length, swing phase, or walking stability, to provide deeper insights. However, this study focused on selected outcomes to emphasize basic gait and balance abilities and provide a comprehensive overview.

Since many outcomes were not normally distributed, the significance of sex-specific correlation differences should be handled with care, but they provide a first impression and direct future focus of sex-specific analysis on walking velocity and gait. To account for the small subgroup sample size and the lack of normal distribution, r_s_ values were reported, and inferential statistics were provided in addition to 95% CIs for a more comprehensive overview.

The results’ transferability to clinical or frail populations must be reviewed with care as only healthy and active participants were included in this study. Finally, this work focused on plantar flexion and dorsiflexion strength, while neglecting the possible influence of inversion and eversion strength, which could explain further balance variance. Furthermore, it cannot be ruled out that the fixed order of exercises might have influenced each other and caused either fatigue or task-learning effects within the test session. Reliability metrics should be included in future studies to prove internal data validity (Warneke et al., 2025).

Due to the high task specificity in strength tests (Warneke et al., 2023a), it could be hypothesized that static balance tasks might show stronger correlations with isometric strength tests. This relationship, however, was not analyzed in this study. This could under- or overestimate the relationship between strength and balance outcomes.

## 5 Conclusion

Maximal plantar flexor and dorsiflexor strength were significantly correlated mostly with dynamic movement and balance tasks measured in this study. The violation of the normal distribution assumption necessitates further research that includes larger samples to counteract individual outliers, while longitudinal research studies are necessary to explore the effects of long-term interventions to confirm the indicated relevance for balance. Although fall observations were not included this study, future research should investigate the role of lower-leg strength as a predictive parameter for developing prevention routines.

## Data Availability

The raw data supporting the conclusions of this article will be made available by the authors, without undue reservation.
